# MiR-27b regulates podocyte survival through targeting adenosine receptor 2B in podocytes from non-human primate

**DOI:** 10.1038/s41419-018-1178-5

**Published:** 2018-11-14

**Authors:** Zuokang Zheng, Hong Hu, Yanrong Tong, Zhixia Hu, Shiyu Cao, Ce Shan, Wenhe Lin, Yike Yin, Zhonghan Li

**Affiliations:** Center of Growth, Metabolism and Aging, Key Laboratory of Bio-Resource and Eco-Environment of Ministry of Education, State Key Laboratory of Biotherapy, College of Life Sciences, Sichuan University, Chengdu, Sichuan 610064 China

## Abstract

MicroRNAs are a group of small non-coding RNAs that play key roles in almost every aspect of mammalian cell. In kidney, microRNAs are required for maintaining normal function of renal cells, disruption of which contributes to pathogenesis of renal diseases. In this study, we investigated the potential role of miRNAs as key regulators of podocyte survival by using a primary cell culture model from non-human primates (NHPs). Through microRNA profile comparison in glomeruli from mouse, rat and NHP, miR-27b was found to be among a list of glomeruli-enriched miRNA conserved across species. In NHP primary podocyte culture, significant downregulation of miR-27b was observed during treatment of puromycin aminonucleoside (PAN), a classic nephrotoxin. Overexpression of miR-27b enhanced PAN-induced apoptosis and cytoskeleton destruction in podocytes while its inhibition had a protective effect. Target identification analysis identified Adora2b as a potential direct target of miR-27b. Ectopic expression of miR-27b suppressed both Adora2b mRNA and protein expression, whereas inhibition of miR-27b increased the transcript and protein expression levels of Adora2B. Dual luciferase assay further confirmed Adora2b as a direct target of miR-27b. Furthermore, knockdown of Adora2b by siRNAs enhanced PAN-induced apoptosis, similar to the phenotypes we had observed with miR-27b overexpression. In addition, stimulating the adenosine signaling by an Adora2b agonist, NECA, improved podocyte survival upon PAN treatment. Taken together, our data identified a novel role of miR-27b-adora2b axis in primary podocyte survival upon injury and suggested a critical role of adenosine signaling pathway in podocyte protection.

## Introduction

Chronic kidney disease (CKD) is a world-wide public health issue, with adverse outcomes of kidney failure^1,2^. Failure to maintain the glomerular filtration barrier directly contributes to the onset of CKD^3^. The visceral epithelial cells, also called podocytes, are crucial for the maintenance of this renal filtration barrier. Direct podocyte injury contributes to the onset and progression of glomerular disease, such as minimal change disease (MCD), focal segmental glomerular sclerosis (FSGS), diabetic nephropathy, and HIV-associated nephropathy (HIVAN)^4^. Therefore, gaining a better understanding of podocytes biology could be of importance to the mechanistic studies of human kidney diseases and development of novel therapeutics and biomarkers.

MicroRNAs (miRNAs) are endogenous ~22 nt RNAs that play important regulatory roles by targeting mRNAs for cleavage or translational repression^5–7^. In kidney, selective disruption of microRNA biogenesis pathways in mouse podocytes led to severe glomerular injury and proteinuria^8–11^, suggesting microRNAs play pivotal roles in maintaining podocyte function. More recently, several microRNAs have been found to regulate various processes in podocytes, such as fibrosis, apoptosis, and glomerulosclerosis. MiR-195 and miR-218 promotes apoptosis of podocytes under high-glucose conditions, whereas miR-23b, miR-21, miR-25, and miR-377 reduces fructose-induced podocyte oxidative stress and inflammation^12–17^. Decreased miR-26a correlates with the progression of podocyte injury in autoimmune glomerulonephritis^18^, miR-29a^19^, and miR-499^20^, respectively, ameliorate hyperglycemia and PAN-induced podocyte dysfunction, although downregulation of miR-30 promotes podocytes injury^21^. However, other microRNA-mediated regulatory pathway in podocytes are still not well understood and of great interest in the field. In this study, through a microRNA profile analysis in glomeruli from mouse, rat, and NHP, we identified miR-27b among a list of conserved microRNAs expressed in glomeruli of different species. In a primary podocyte model from NHP (Cynomolgous Macaque), miR-27b was found to be strongly downregulated upon induction of podocyte injury by puromycin aminonucleoside (PAN), a classic nephrotoxin. Overexpression of miR-27b enhanced PAN-induced cell death by increasing caspase activity and destruction of cytoskeleton structures, whereas inhibiting miR-27b showed protective effects. Target analysis and dual luciferase assay revealed that adenosine receptor 2b (Adora2b, A2B) was one of the key direct targets of miR-27b, which was significantly upregulated upon PAN treatment in both rat and NHP podocytes. Knockdown of A2B enhanced PAN-induced cell death, similar to miR-27b overexpression while stimulating its activity by NECA had protective effects. In summary, we have identified a novel miR-27b-Adora2b axis regulated podocyte survival upon injury and provided evidence that adenosine signaling pathway may be a potential therapeutic target for podocyte protection.

## Results

### miR-27b is a conserved glomeruli-enriched miRNA across species

**Fig. 1:**
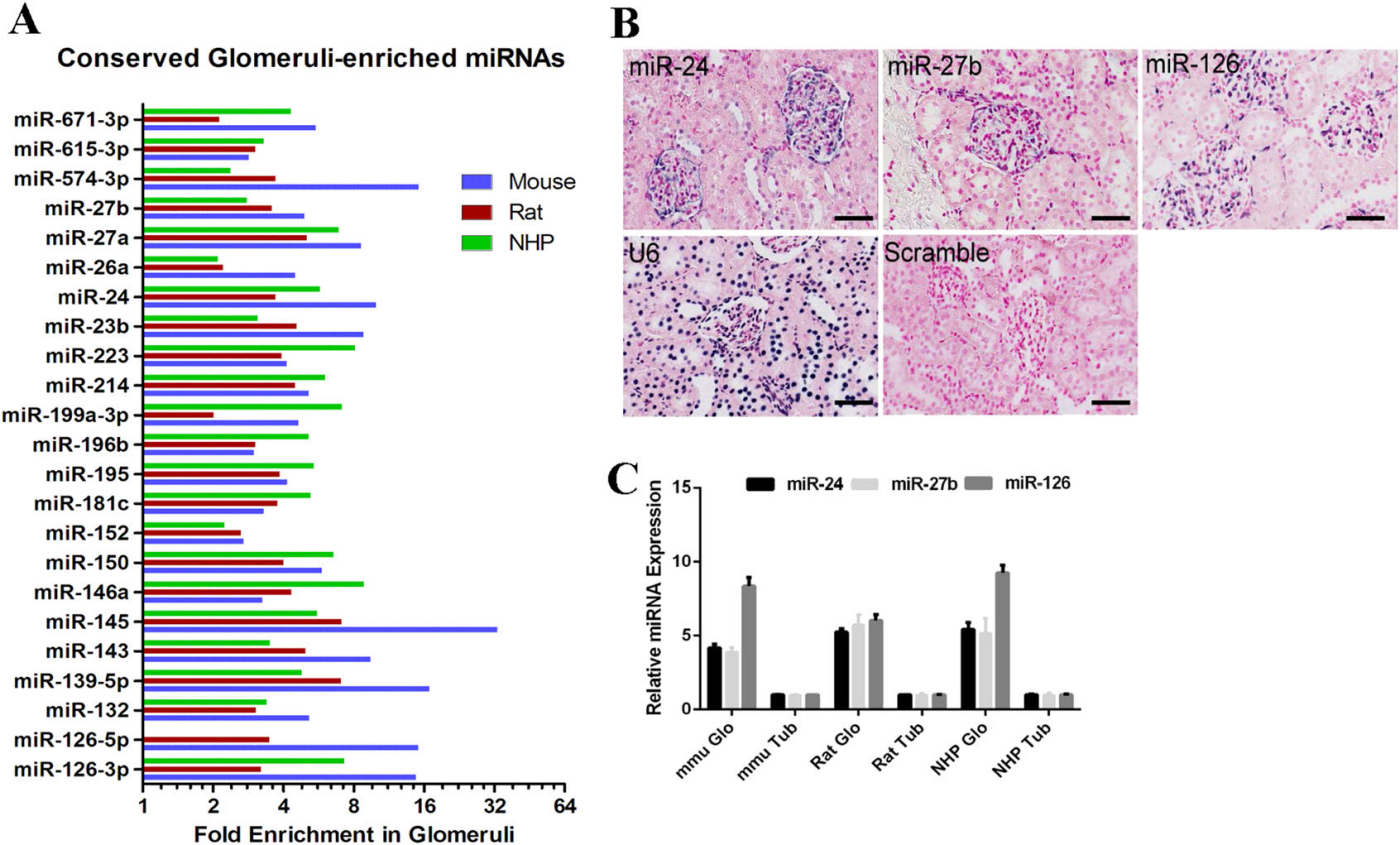
Identification and validation of glomeruli-enriched microRNAs. **a** A group of microRNAs was identified to be conservatively expressed in glomeruli from mouse, rat, and NHP by TaqMan Microarray. **b** miR-24, 27b, and 126 were confirmed to be enriched in glomeruli of Sprague Dawley rats by in situ hybridization. FFPE sections of normal rat kidney tissues were stained with 40 nM microRNA probe, 1 nM U6 probe, and 40 nM Scramble control. Scale bar: 50 µm. **c** miR-24, 27b, and 126 were also enriched in NHP glomeruli. Glomeruli and tubule tissues from Sprague Dawley rats (*n* = 3) and NHP kidneys (*n* = 2) were collected for RNA extraction and qPCR analysis of miRNA expressions. Error bar represents data from 2 to 3 animals and two technical replicate samples per animal. Glo glomeruli; Tub ubule

As previously mentioned, podocyte-specific loss-of-miRNAs resulted in significant proteinuria and glomerular injury^8–11^, suggesting that microRNAs are critical to maintain the glomerular filtration barrier. However, only a limited set of miRNAs have been shown to regulate different processes of podocyte biology^12–19,21^. To investigate whether there are previously unappreciated microRNA involved in the biology of podocyte, we did a microRNA profile in glomeruli from mouse, rat, and NHP, and identified a series of conserved microRNAs expressed in glomeruli (Fig. 1a). Among them, miR-27b was one of the most interesting microRNAs to us, as it was less studied in podocyte biology and it was expressed in a cluster of miRs, including miR-24 and miR-23b. MiR-24 antagonism had a protective effect on renal ischemia reperfusion injury^22^, whereas miR-23b alleviated kidney fibrosis and proteinuria by targeting G3BP2^17,23^. Therefore, we hypothesized that miR-27b might also have an important role in renal cell biology. We first confirmed that miR-27b was indeed expressed specifically in glomeruli by LNA based in situ hybridization (Fig. 1b). Two previously confirmed glomerular miRNAs, miR-24^9^, and miR-126^8^ were also included as positive controls in our ISH experiments. To further confirm whether miR-27b expression was conserved across species, we isolated both glomeruli and tubular tissues from Sprague Dawley rats and NHP animals. RT-qPCR analysis indicated that miR-27b was 4 ~ 5-fold higher expressed in glomeruli than in tubular tissues (Fig. 1c) and its glomerular enrichment was well conserved among mouse, rats and non-human primates. Together, our data suggested miR-27b was indeed a glomeruli-enriched miRNA expressed in multiple species.

### Primary podocytes serve as an in vitro model for PAN-induced injury

**Fig. 2:**
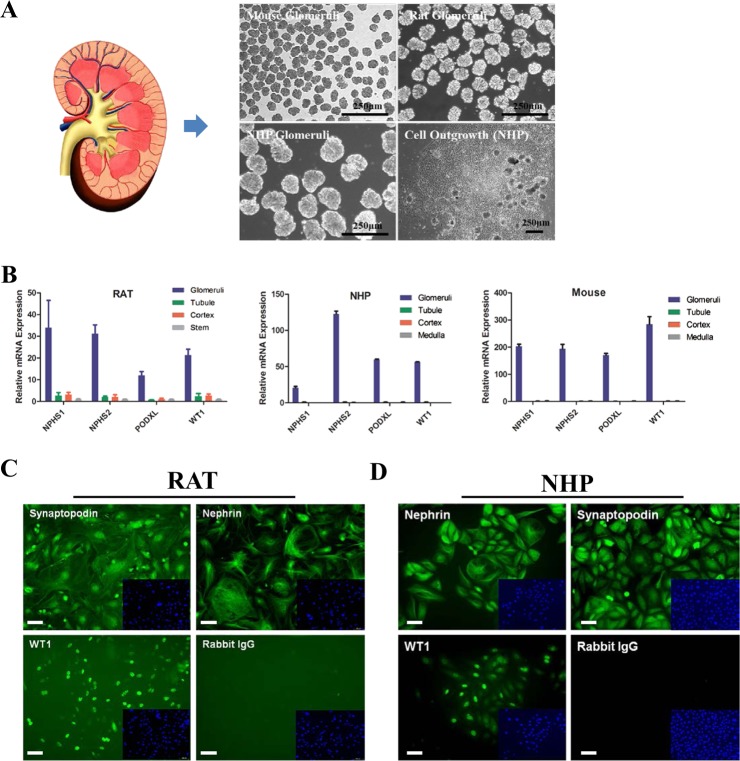
Isolation and characterization of primary podocytes from NHPs. **a** The glomeruli from different mammalian species were isolated by classical sieving techniques. Scale bar: 250 µm. NHP glomeruli were isolated through 160 µm and 100 µm sieves. Rat glomeruli were isolated through 100 µm and 75 µm sieves, whereas mouse ones were isolated using 75 µm and 40 µm sieves. **b** Isolated glomeruli tissues have high expression of published glomerular marker genes. Marker genes were analyzed in isolated tissues, including glomeruli, tubule, kidney cortex, and medulla. Error bar represents data from duplicate wells from mixed samples of 2–3 animals. **c** Primary rat podocytes were positive for traditional podocyte markers. The glomeruli were isolated and plated onto collagen coated plates followed by an outgrowth of arborized mature podocytes that stain positive for well-characterized podocyte markers, including Wilms Tumor 1 (WT-1), Synaptopodin, and Nephrin. Small inserts represent nuclear staining by DAPI. Scale bar: 50 µm; **d** Primary NHP podocytes also stained positive for podocyte markers. Scale bar: 50 µm

Although established immortalized podocyte cell lines have been widely used and led to many discoveries in understanding basic biology of renal cells^24–27^, inherited disadvantages are also of concern such as low expression of nephrin and other primary renal cell markers, difficulty in culture under selective temperature etc^25,28,29^. Thus, we decided to use primary podocytes, especially NHP ones, as an in vitro model to investigate microRNA function. We employed a modified version of the protocol established by Holdsworth’s protocol to isolate glomeruli from Cynomolgus Macaque monkeys by using a two-sieve procedure (Materials and Method)^30^. The purity of isolated NHP glomeruli was >95% (Fig. 2a) and similar results were also achieved using rat and mice kidneys (Fig. 2a). To further confirm the purity of the isolated glomeruli, we investigated the expression of glomerular-specific genes in the isolated cultures. We extracted total RNAs from isolated glomeruli, as well as tubular tissues, the cortex and medulla and RT-qPCR analysis was performed to evaluate the expression of established glomerular marker genes, including NPHS1, NPHS2, PODXL, and WT1^31^. Indeed, all these genes were highly enriched in isolated glomeruli with only minimal detection in other tissues (Fig. 2b), suggesting NHP glomeruli can be efficiently isolated using the modified procedure.

**Fig. 3:**
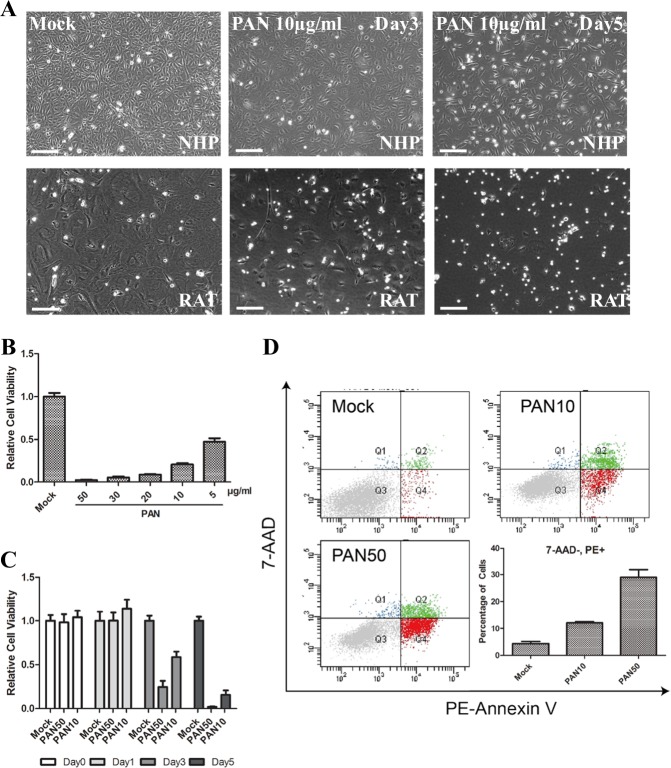
Use of primary podocytes from NHPs as an in vitro model for PAN-induced cytotoxicity. **a** PAN-induced dose-dependent cell death in primary NHP and rat podocytes. NHP podocytes were treated with 10 µg/ml of PAN for 3–5 days before taking images. Rat podocytes were treated with 10 µg/ml of PAN for 3–5 days before taking images. Scale bar: 150 µm. **b** NHP podocytes responded to PAN in a dose-dependent manner. Cells were treated with PAN at different concentrations and collected at day 5 for cell viability test. Error bar represents data from eight biological replicates. **c** NHP podocytes responded to PAN in a time-dependent manner. Cells were treated with PAN at 50 or 10 µg/ml and collected at different time points for cell viability test. Error bar represents data from eight biological replicates. **d** Analysis of PAN-induced toxicity in NHP podocytes using Annexin V/7-AAD staining. NHP podocytes were treated with 10 or 50 µg/ml PAN for 3 days before collected for FACS analysis. PAN10:10 µg/ml, PAN50: 50 µg/ml. Error bar represents data from three technical replicates

To harvest podocytes, we seeded the purified glomeruli on collagen coated plates without any enzyme digestion, which can avoid impairing cell viability and liberating mesangial cells^32^. In about 1–2 weeks, ~20% glomeruli attached to the culture plates and produced outgrowth of cobblestone-like cells. Surprisingly, NHP podocytes can be passaged for 2–3 times before they stop proliferating, enabling us to carry out large-scale-cell biology experiments with good consistence. In comparison and in agreement with previously published work, subcultures of similarly isolated rat podocytes result in arborized cells, however the proliferation capability is lost^28^. To further characterize the cells and ensure their podocyte identity, we did immunostaining analysis of known podocyte markers on these cells, with primary rat podocytes as the control. Almost all the NHP cells were positive for Nephrin and Synaptopodin immunostaining and ~70% of them were also WT1 positive (Fig. 2c, d), supporting their podocyte identity. We further tested their response to a known nephrotoxin, puromycin aminonucleoside (PAN) and found them sensitive to PAN treatment even at lower doses (10 µg/ml) and exhibited significant cell death upon treatment (Fig. 3a), which was also shown in control rat podocytes (Fig. 3a). Dose- and time-dependent evaluation of PAN-induced injury in NHP podocytes all indicated that these primary cells responded strongly to the nephrotoxin treatment (Fig. 3b, c). In addition, the cell death induced by PAN was apparently through apoptotic pathway activation, as we detected an increase of Annexin V positive cells upon PAN treatment (Fig. 3d). Together, our data suggest that the primary podocytes isolated from NHP glomeruli maintained their identity and responded well to the known nephrotoxin, PAN. Given the large number of cells we were able to obtain from each NHP kidney, we decided to use these primary cells as an in vitro system to study podocyte function, especially podocyte-specific miRNAs and their response to drug-induced injury.

### miR-27b regulated PAN-induced apoptosis in NHP podocytes

**Fig. 4:**
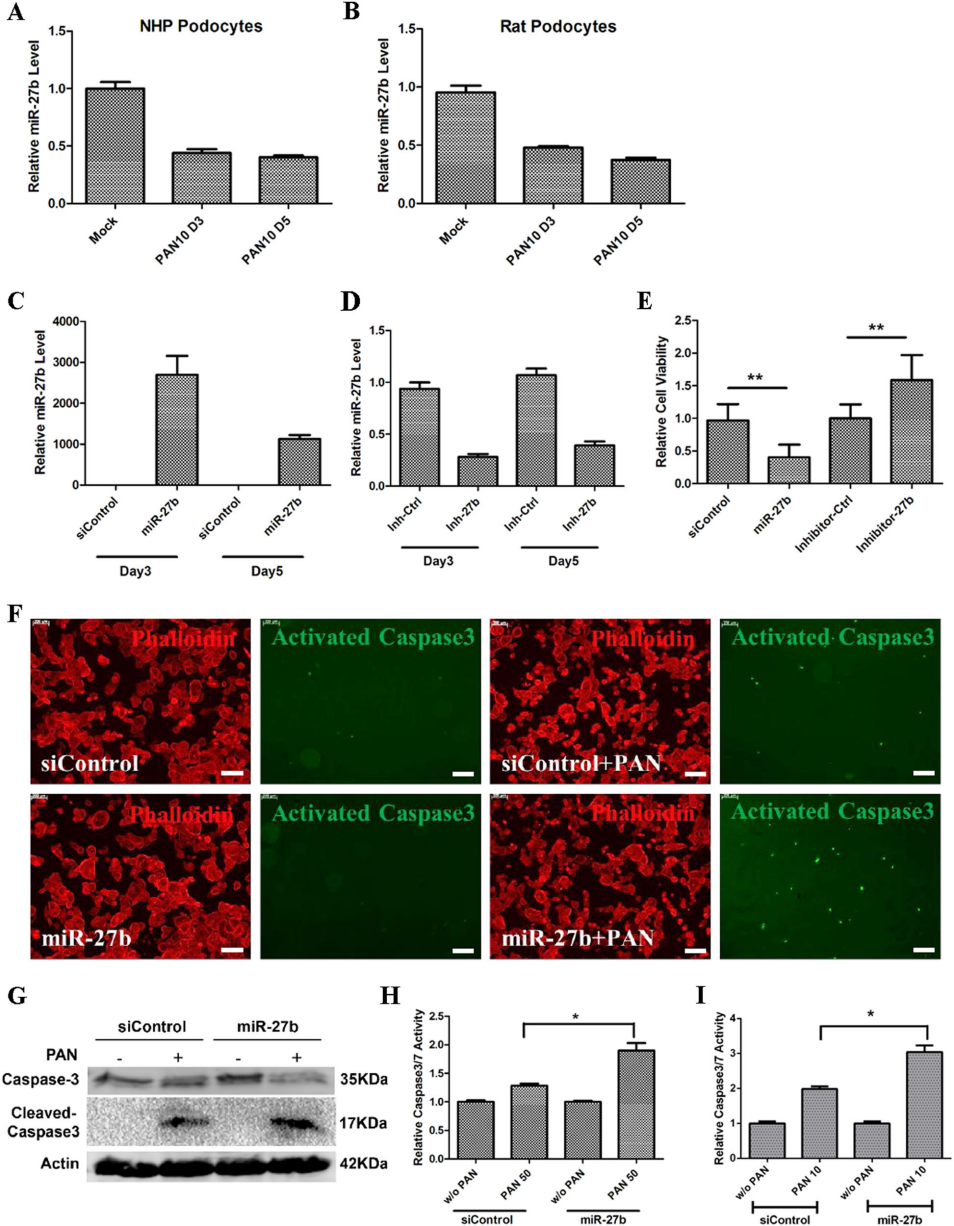
MiR-27b regulated PAN-induced apoptosis in NHP podocytes. **a** miR-27b was significantly suppressed upon PAN treatment. Cells were exposed to 10 µg/ml PAN for 3–5 days before collected for RT-qPCR. Relative miR expression was calculated using U6 as normalization control. Error bar represents data from two independent experiments. **b** PAN-induced miR-27 change was also observed in primary rat podocytes. Cells were treated with PAN for 3 and 5 days before collected for miR-qPCR analysis. Error bar represents data from two independent experiments. **c** miR-27b was efficiently transfected into NHP podocytes. 50 nM miRNA mimic and siControl were transfected into NHP podocytes using Lipofectamine RNAiMax. Cells were collected at day 3 and day 5 post transfection and miRNA expression was analysis using miR-X kit (Clontech). Error bar represents data from two independent experiments. Data were normalized to U6. **d** Endogenous miR-27b was efficiently knocked-down by transfecting miR-27b hairpin inhibitor into NHP podocytes. miR inhibitor control and miR-27b inhibitor were transfected into NHP podocytes at the final concentration of 50 nM. Cells were collected for miRNA qPCR at day 3 and day 5 post transfection. Data were normalized to U6. Error bar represents data from two independent experiments. **e** miR-27b overexpression enhanced PAN-induced cell death while miRNA inhibition led to increased cell viability. Cells were transfected with 50 nM miRNA mimic or inhibitor and treated with 10 µg/ml PAN for 5 days before collected for cell viability test using CellTiter 96 kit (Promega). Relative cell viability was calculated by normalizing absorbance readings from PAN-treated samples to non-treated controls. Error bar represents data from three independent experiments with eight replicates. **p < 0.01 by Student's *t*-test. **f** miR-27b overexpression led to increased caspase3 activation. Cells were transfected with 50 nM miRNA mimic and treated with 50 µg/ml PAN for 36 h before fixation and immunostaining. Scale bar: 200 µm. **g** Cleaved caspase-3 expression was increased by miR-27b overexpression in NHP podocytes. Cells were treated with 50 µg/ml PAN for 48 h before collected for active caspase3 by western blotting. Actin was used as the loading control. **h** miR-27b overexpression led to increased caspase3/7 activation. Cells were treated with 50 µg/ml PAN for 36 h before collected for /7 glo assay (Promega). Error bars represent data from eight replicates. *p < 0.05 by Student's *t*-test. **i** miR-27b overexpression led to increased caspase3/7 activation. Cells were treated with 10 µg/ml PAN for 36 h before collected for /7 glo assay (Promega). Error bars represent data from eight replicates. *p < 0.05 by Student's *t*-test

Next we investigated whether miR-27b expression was altered with podocyte injury. PAN treatment in NHP podocytes resulted in a decrease of miR-27b expression (Fig. 4a). A similar result was also observed in primary rat podocytes (Fig. 4b), suggesting that a conserved mechanism might exist in the regulation of miR-27b. To manipulate the expression of miR-27b in the primary cells, we transfected them with its specific mimic and hairpin inhibitors. Liposome-mediated transfection efficiently delivered both miR-27b mimic and inhibitors into the cells and the expression was maintained for at least 5 days (Fig. 4c, d). We then investigated whether overexpression or inhibition of miR-27b in NHP podocytes could affect PAN-induced injury. Interestingly, compared with siControl transfected cells, transfection of miR-27b enhanced PAN-induced cell death in NHP podocytes, whereas its inhibition attenuated PAN’s toxicity (Fig. 4e). To further confirm this, immunostaining of activated, a marker for apoptosis, was carried in both non-treated and PAN-treated primary podocytes and the protein level of activated was also detected by western blotting. Overexpression of miR-27b indeed resulted in increased activation of Caspase3 (Fig. 4f, g). In addition, Caspase3/7 activity was also significantly increased in miR-27b mimic transfected cells, and observed with two different doses, 50 µg/ml (Fig. 4h) and a lower 10 µg/ml dose (Fig. 4i). Notably, PAN treatment at 50 µg/ml only showed small increase of Caspase3/7 activity, possibly due to strong immediate cell loss upon the treatment (Fig. 3c). Together, our data indicated that a feedback downregulation of miR-27b existed in NHP podocytes as their response to PAN-induced injury and manipulating miR-27b expression could have a significant impact on podocyte survival.

### miR-27b regulated PAN-induced damage of cytoskeleton

**Fig. 5:**
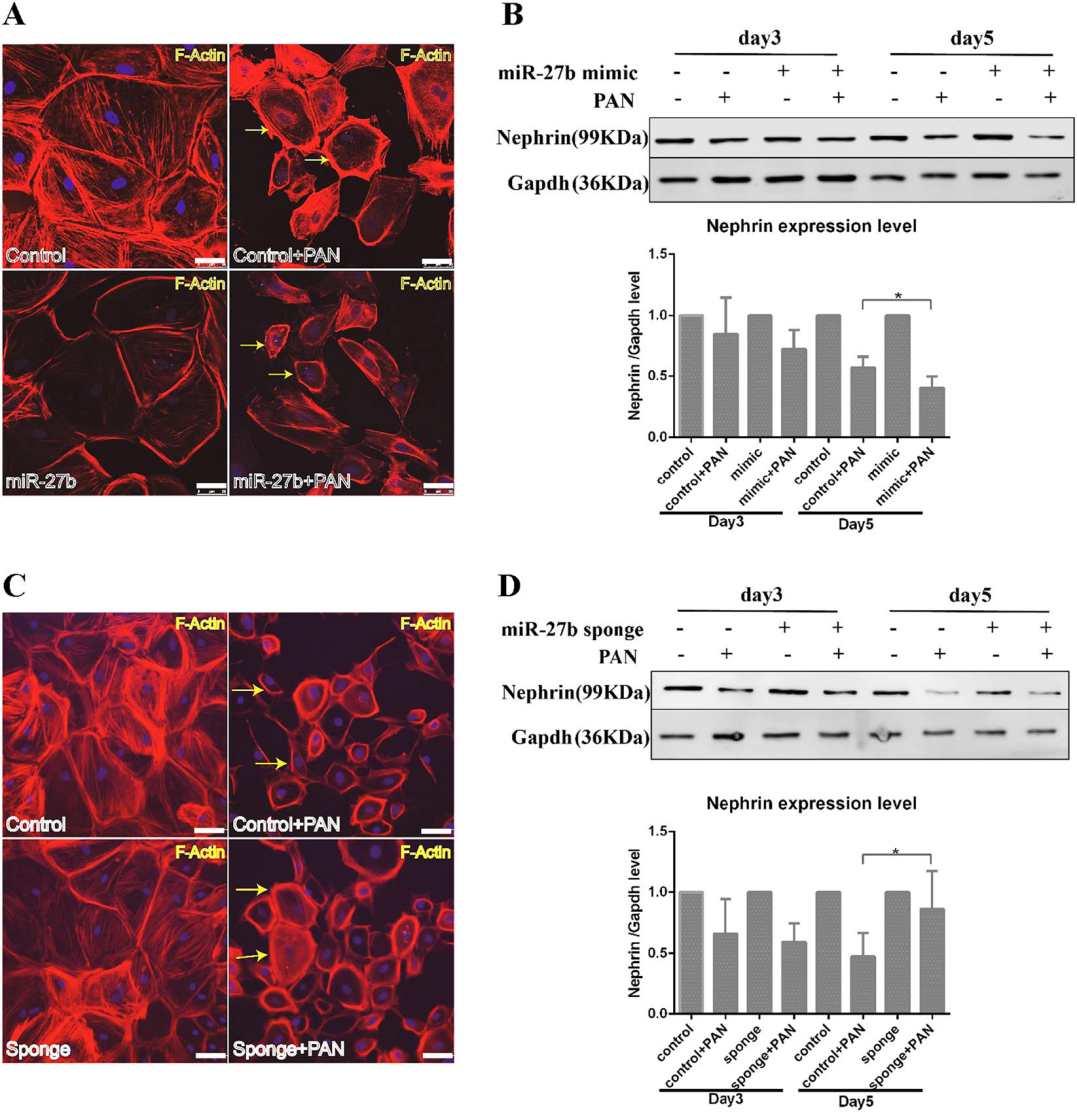
miR-27b regulated PAN-induced damage of cytoskeleton. **a** miR-27b overexpression enhanced PAN-induced cytoskeleton destruction. Cells were transfected with 50 nM miRNA mimic and treated with 10 μg/ml PAN for 3 days before fixation and staining. Scale: 50 µm. **b** Protein expression of nephrin was decreased by miR-27b. Cells were transfected with 50 nM miRNA mimic and treated with 10 µg/ml PAN for 3–5 days before collected and protein expression of nephrin was analyzed by western blotting. GAPDH was used as the loading control. **c** Inhibit the function of miR-27b by sponge ameliorated cytoskeleton damage induced by PAN. Scale bar: 50 µm. **d** Protein expression of nephrin was reversed by miR-27b sponge. Error bars represent data from three independent experiments. *p < 0.05 by Student's *t*-test

The specialized morphology of different mammalian cell types reflects their unique physiological functions. The glomerular podocyte are composed by a cell body, major processes, and foot processes (FPs)^33,34^, which are interconnected actin cytoskeleton structure. The spaces between the foot processes of neighboring podocytes form filtration slits that are bridged by specialized intercellular junctions known as slit diaphragms. In addition, the transmembrane protein Nephrin is also expressed at the intercellular junction of podocytes^35,36^. Therefore, we would like to investigate whether overexpression or inhibition of miR-27b in NHP podocytes could affect PAN-induced damage of cytoskeleton. Interestingly, compared with siControl transfected cells, transfection of miR-27b enhanced PAN-induced cell morphology change in NHP podocytes (Fig. 5a), and the expression of Nephrin was significantly decreased (Fig. 5b). Although its inhibition by a miR-27b decoy attenuated PAN’s effect on morphology change (Fig. 5c) and the expression of Nephrin was increased compared to control group (Fig. 5d). Together, our data indicated that manipulating miR-27b expression could have an impact on podocyte cytoskeleton change upon PAN treatment.

### Adora2b is a direct target of miR-27b in NHP podocytes

**Fig. 6:**
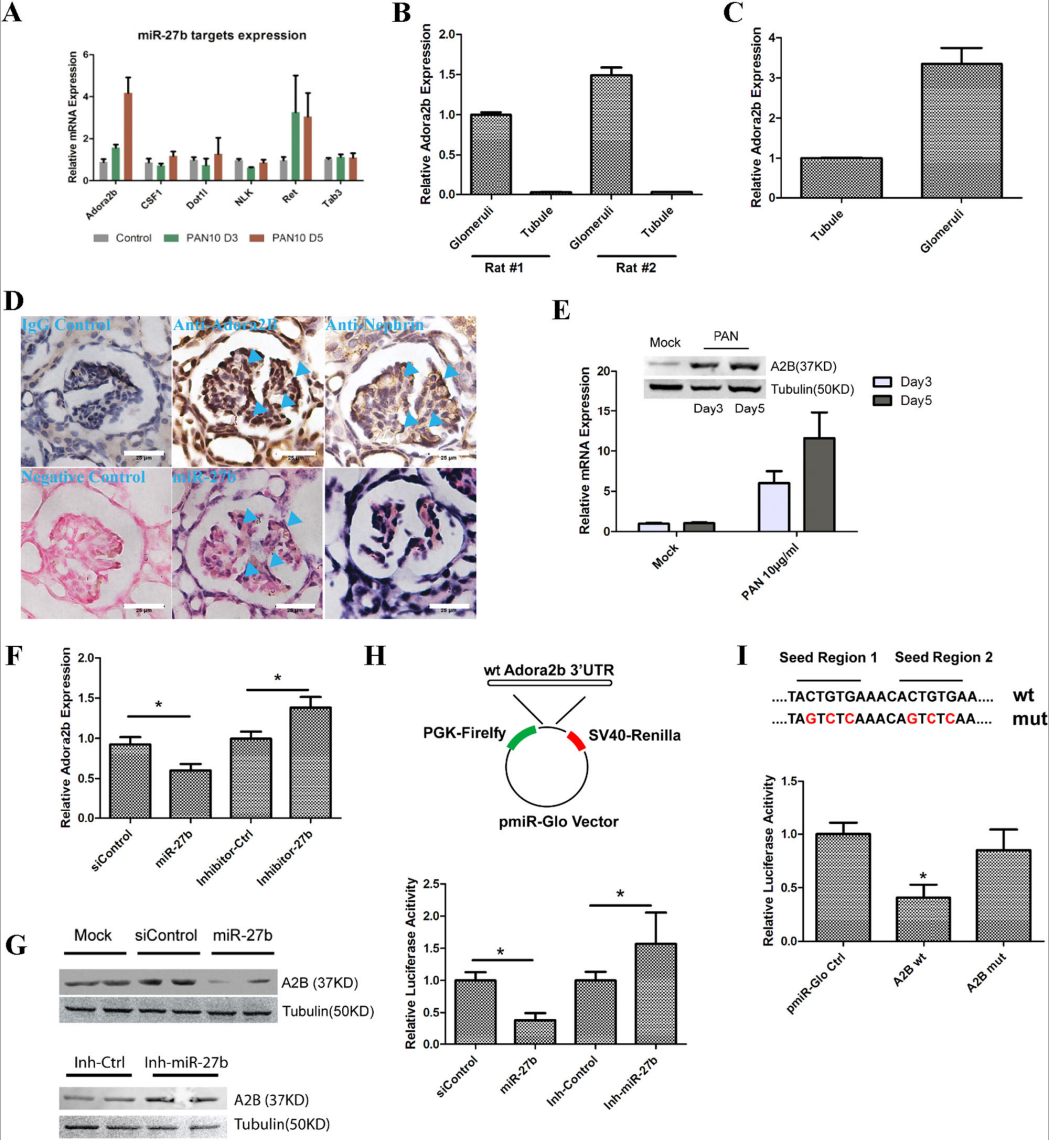
Adora2b is a direct target of miR-27b in NHP podocytes. **a** Adora2b was induced upon PAN treatment in primary rat podocytes. Rat podocytes were treated with 10 µg/ml PAN for 3 and 5 days before collected for RT-qPCR. Several predicted miR-27b targets from TargetScan with multiple conserved miRNA targeting sites were selected for the analysis. Error bar represents data from two independent experiments. **b** Adora2b was specifically expressed in rat glomeruli. Primary tissues of glomeruli and tubule were isolated from two different rats. Total RNAs were extracted and expression of Adora2b was analyzed by RT-qPCR. Data were normalized to GAPDH. Error bar represents data from two technical replicates. **c** Adora2b was enriched in NHP glomeruli. Expression of Adroa2b was analyzed using primary tissues from NHP. Error bar represents data from two technical replicates. **d** Adora2b and miR-27b were confirmed to be expressed in same glomeruli of NHP by immunohistochemistry and in situ hybridization. FFPE sections of normal monkey kidney tissues were stained with anti-Adora2B and anti-Nephrin antibodies as well as 40 nM microRNA probe. The arrow indicates the same site of Adorae2b, Nephrin, and miR-27b. Scale bar: 25 µm. **e** Adora2b expression was strongly induced by PAN treatment in NHP podocytes. NHP podocytes were treated with 10 µg/ml PAN for 3–5 days before collected. Upper: protein expression was analyzed by western blotting. Lower: mRNA expression was analyzed by RT-qPCR. Error bar represents data from two independent experiments. **f** mRNA expression of Adora2b was suppressed by miR-27b in NHP podocytes. Cells were transfected with 50 nM of siRNAs such as siControl/miR-27b mimic, Inhibitor control/miR-27b inhibitors. Total RNAs were extracted for RT-qPCR analysis at 48 h after transfection. Error bar represents data from three independent experiments. *p < 0.05 by Student's *t*-test. **g** Protein expression of Adora2b was suppressed by miR-27b in NHP podocytes. Cells were transfected the same way as in **f** and protein expression of Adora2b was analyzed 48 h post transfection by western blotting. Tubulin was used as the loading control. **h** Adora2b is a direct target of miR-27b. Adora2b 3′-UTR was cloned into pmiR-glo luciferase reporter (Promega). Dual luciferase assay was done in 293T cells with both reporter and miRNA transfection. miRNA mimic or inhibitor were transfected at 50 nM for 2 days and luciferase activity was analyzed using Dual-Glo assay system (Promega). Error bar represents data from two independent experiments with eight replicates. *p < 0.05 by Student's *t*-test. **i** Seed region mutations abolish miR-27b’s suppression on Adora2b. Three point-mutations were introduced into the seed region of each miR-27b target site. These luciferase reporters were then transfected into 293T cells and analyzed in the same way as in **g**. Error bar represents data from two independent experiments with eight replicates

To investigate the molecular mechanism for miR-27b-mediated regulation of podocyte survival and cytoskeleton changes upon PAN treatment, we sought to identify the target gene regulated by miR-27b in NHP podocytes. As the regulation of miR-27b expression was conserved between rats and NHPs (Fig. 4a, b), we reasoned that the target gene of miR-27b must be also conserved. We then analyzed potential target genes predicted by TargetScan. Although there are about 900 conserved targets predicted by TargetScan, only about 70 genes have multiple conserved sites in their 3′-UTR. We reasoned that genes with more target sites could be preferably targeted by miR-27b when expressed at similar levels^37^. Thus, we focused our efforts on the 70 genes with at least two conserved miR-27b target sites. Out of the 70 genes, only six have been documented with any type of kidney development or disease, including Adora2b (A2B), CSF1, Dot1l, Nlk, Ret, and Tab3. To investigate whether these predicted genes could be the true targets of miR-27b, we first analyzed their mRNA expression in primary rat podocytes with and without PAN treatment. As mRNA destabilization is the main effect of miRNA-mediated translational repression^38^, we reasoned that as miR-27b was downregulated upon PAN treatment (Fig. 4a, b), its target genes should be released/increased upon PAN treatment. Indeed, RT-qPCR analysis revealed that out of the six candidate genes, only Adora2b was induced upon PAN treatment in rat podocytes in a time-dependent manner (Fig. 6a). In addition, gene expression analysis using isolated glomeruli and tubular tissues confirmed that Adora2b was expressed specifically in rat glomeruli (Fig. 6b). We then moved to confirm if these observations were also conserved in non-human primates. Indeed, Adora2b was also enriched in NHP glomeruli (Fig. 6c). Moreover, ISH and IHC analysis indicated that Adora2b expressing cells also had miR-27b expression, as well as other traditional podocyte markers, such as Nephrin (Fig. 6d). Expression of both mRNA and protein of Adora2b in NHP podocytes were highly induced upon PAN treatment (Fig. 6e). To test whether miR-27b could regulate Adora2b’s expression, miR-27b mimic and inhibitors were transfected in NHP podocytes and Adora2b mRNA expression was analyzed by RT-qPCR. MiR-27b mimic transfection resulted in ~40% decrease in Adora2b mRNA expression, whereas its inhibition increased Adora2b expression by ~30–40% (Fig. 6f). Western blot analysis also confirmed that miR-27b mimic transfection significantly reduced Adora2b’s protein expression while miR-27b inhibition upregulated Adora2b (Fig. 6g). To ensure Adora2b was directly targeted by miR-27b, we cloned the 3′-UTR of NHP Adora2b gene into pmiR-glo dual luciferase reporter. We then assayed luciferase expression upon miR-27b mimic and inhibitor transfection in 293FT cells. MiR-27b mimic significantly reduced firefly luciferase expression compared with siControl, although miR-27b inhibition increased luciferase expression (Fig. 6h). As seed regions of miRNAs largely determined the target specificity, we introduced a few point mutations in miR-27b target sites to disrupt potential binding with miR-27b. Whereas wild-type luciferase reporter could be efficiently repressed by miR-27b, luciferase expression of the mutant vector was released from miR-27b mediated repression (Fig. 6i). Together, our data suggested that Adora2b was induced in PAN-treated NHP podocytes and was a direct target of miR-27b.

### Stimulating adenosine signaling through adora2b ameliorated PAN-induced injury in NHP podocytes

**Fig. 7:**
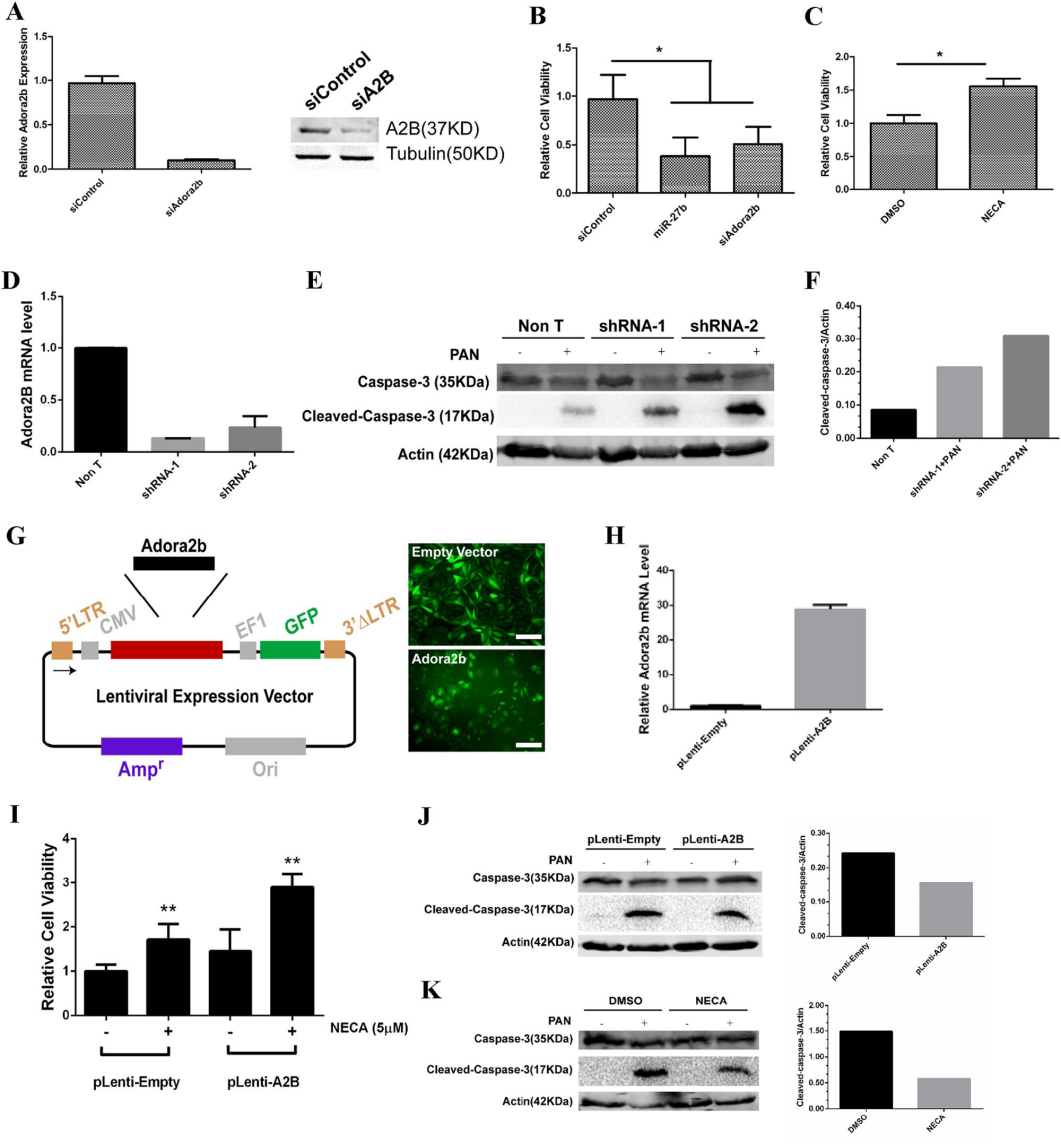
Stimulating Adenosine signaling through Adora2b reversed PAN-induced injury in NHP podocytes. **a** Adora2b was efficiently knocked-down by siRNAs in NHP podocytes. A total of 50 nM siRNAs were transfected into the cells using Lipofectamine RNAiMax (Life Technologies). Total RNAs were isolated for RT-qPCR at 48 h post transfection. Data were normalized to GAPDH. Error bar represents data from two replicates (Left panel). Protein expression of Adora2b was also significantly decreased in siA2B transfected cells (Right panel). Total proteins were isolated for western blotting at 48 h post transfection. Tubulin was used as the loading control. **b** Knockdown of Adora2b promoted PAN-induced injury in NHP podocytes. Cells were transfected with siControl, miR-27b mimic, and siA2B at the final concentration of 50 nM. PAN treatment (10 µg/ml) was initiated 1 day after transfection. Cells were subject to viability test using CellTiter-96 (Promega) at day 5 post PAN treatment. Relative cell viability was calculated by normalizing absorbance readings from PAN-treated samples to non-treated controls. Error bar represents data from two independent experiments with eight replicates. *p < 0.05 by Student's *t*-test. **c** Stimulating adora2b had a protective role in PAN-induced injury in NHP podocytes. Cells were stimulated with 5 µM NECA and PAN treatment (10 µg/ml) was initiated 1 day after. Cell viability test was the same as in **b**. Error bar represents data two independent experiments with eight replicates. *p < 0.05 by Student's *t*-test. **d** Adora2b was efficiently knocked-down by shRNAs in NHP podocytes. Total RNAs were isolated for RT-qPCR at 72 h post lentivirus infection. Data were normalized to GAPDH. Error bar represents data from two technical replicates. **e** Protein expression of Cleaved Caspase-3 was elevated in shRNA infected NHP podocytes cells. Cells were infected with shRNA and PAN treatment (50 µg/ml) was initiated 1 day after. Total proteins were isolated for western blotting at 48 h post PAN treatment. Actin was used as the loading control. **f** Quantification of cleaved caspase-3 expression. Relative quantification was performed by normalizing signal of cleaved caspase-3 to actin. **g** Overexpression of Adora2b by lentiviral vectors. Coding sequence of Adora2b was cloned into pCMV-MCS-EF1-copGFP vector (System Biosciences). Lentivirus transduced cells were spotted by GFP imaging. Scale: 100 µm. **h** Overexpression of Adora2b by lentiviral vectors. Total RNAs were isolated for RT-qPCR at 72 h post lentivirus infection. Data were normalized to GAPDH. Error bar represents data from two technical replicates. **i** Overexpression of Adora2b had protective effects against PAN-induced injury in NHP podocytes. Equal number of empty or Adora2b transduced cells were seeded in 96-well plates and treated with 10 µg/ml PAN and with or without NECA (5 µM). Data represent two independent experiments with eight replicates. **p < 0.01 by Student's *t*-test. **j** Protein expression of Cleaved Caspase-3 was modestly suppressed in pLenti-A2B infected NHP podocytes cells (Left panel). Cells were infected with empty or Adora2b and PAN treatment (50 µg/ml) was initiated 1 day after. Total proteins were isolated for western blotting at 48 h post PAN treatment. Actin was used as the loading control. Relative quantification was performed for western results (Right panel). **k** Stimulation of Adora2b by NECA decreased the expression of Cleaved Caspase-3 in NHP podocytes (Left panel). Cells were stimulated with 5 µM NECA and PAN treatment (50 µg/ml) was initiated 1 day after. Total proteins were isolated for western blotting at 48 h post PAN treatment. Actin was used as the loading control. Relative quantitation was performed for western results (Right panel)

After confirming that Adora2b is a direct target of miR-27b, we sought to investigate whether miR-27b’s effects are mediated through Adora2B expression upon PAN treatment. We used RNAi approach to knockdown Adora2b expression in NHP podocytes and achieved efficient knockdown of both mRNA and protein expression (Fig. 7a). We then investigated whether knockdown of Adora2b would have similar phenotype as miR-27 upregulation. Indeed, Adora2b knockdown resulted in significant loss-of-cell viability in NHP podocytes upon PAN treatment, which was similar to miR-27b mimic transfection (Fig. 7b). Meanwhile, stimulating Adenosine signaling pathway by a pan-Adora2 receptor agonist, NECA, had the opposite effect and could significantly attenuate PAN-induced apoptosis (Fig. 7c). To further demonstrate that Adora2B was involved in the process of PAN-induced apoptosis of NHP, we constructed two shRNA to knockdown Adora2b (Fig. 7d) and found Adora2b knockdown resulted in increase of activated Caspase3 expression (Fig. 7e, f). To further confirm our findings, we cloned *Adora2b* gene into a lentiviral expression system and overexpressed it in NHP podocytes (Fig. 7g, h). Overexpression of Adora2b itself exhibited only modest protective effects on PAN-induced cell death, however, when combined with NECA stimulation, Adora2b overexpressed cells had significantly higher cell viability compared to empty vector transduced controls (Fig. 7i). The expression of apoptosis marker, cleaved caspase-3, was also lower in Adora2b overexpression and NECA treated cells (Fig. 7j, k). Thus, our data suggest that stimulating adenosine signaling pathway through Adora2b improved podocyte survival upon PAN-induced toxicity and miR-27b regulates podocyte survival through inhibiting Adora2b expression.

## Discussion

**Fig. 8:**
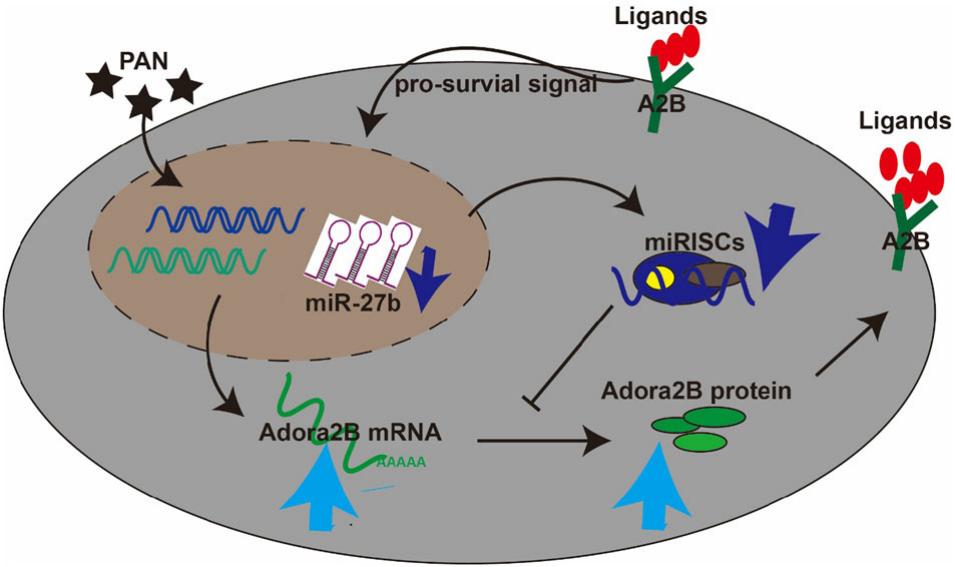
Model of miR-27b-mediated regulation of podocyte survival. Expression of miR-27b is subject to a feedback regulation mechanism induced by PAN treatment in primary podocytes.Decreased expression of miR-27b leads to the released suppression of adenosine receptor 2b expression (A2B). Abundant A2B on the cell surface promotes intracellular pro-survival signaling pathways

In this study, we demonstrate that a novel role of miR-27b-adora2b axis in regulating podocyte survival upon PAN treatment. MiR-27b was conservatively expressed in the glomeruli of mouse, rat, and NHP. A negative feedback regulation of miR-27b expression existed upon PAN treatment. Overexpression of miR-27b enhances the PAN-induced cell death and destruction of cytoskeleton structure in primary NHP podocytes. Conversely inhibiting miR-27b showed protective effects. Target analysis and dual luciferase assay revealed that Adora2b was one of the direct key targets of miR-27b, and overexpression or inhibiting the function of A2B has the similar effect as miR-27b. Together, these data provide evidence that miR-27b-Adora2 axis play an important role in regulating podocyte survival and cytoskeleton integrity upon PAN-induced injury (Fig. 8).

Several miRNAs have been previously reported to be involved in both normal and pathological kidney function. For example, miR-195 and miR-218 promotes podocyte apoptosis under high-glucose conditions, whereas miR-377 reduces fructose-induced podocyte oxidative stress and inflammation^12–14^. Decreased miR-26a correlates with the progression of podocyte injury in autoimmune glomerulonephritis, miR-29a ameliorates hyperglycemia-induced podocyte dysfunction and downregulation of miR-30 promotes podocytes injury^18,19,21^. More recently, miR-27a was found to promote tubular fibrosis^39^ and enhance podocyte depletion in diabetic rats by targeting PPARgamma^40^. Our study added a nice page to this growing list of miRNAs by identifying that miR-27b regulates both podocyte survival and its cytoskeleton integrity upon injury, potentially by targeting adenosine receptor Adora2b, which is a novel reported target of this miRNA.

Adenosine receptors are a group of purinergic G protein-coupled receptors. In human, there are four types of adenosine receptors, called Adora1(A1), Adora2A (A2A), Adora2B (A2B), and Adora3 (A3). Among them, A1 and A3 are inhibitory and suppress adenylyl-cyclase while A2A and A2B are stimulatory and lead to increased cAMP levels^41^. Both A2A and A2B seems to have protective roles in a number of conditions, including ischemia/reperfusion-induced cardiac injury^42,43^ and acute lung injury^44^. In kidney, A2A and A2B were not only involved in regulating physiological activities such as proton secretion in medulla cells^45^, but also had protective effects upon kidney injury^46,47^. Our study demonstrated that Adora2B may be part of a feedback regulatory pathway in regulating podocyte survival and cytoskeleton integrity upon PAN-induced injury, as silencing of Adora2B markedly promoted the apoptosis and cytoskeleton destruction of podocyte.

Finally, podocytes has become one of the key targets in the development of therapeutic strategies for chronic kidney diseases^48^. Rather than immortalized cell line, our results indicate that primary podocytes from NHP may be a valuable in vitro system for mechanistic study of basic podocyte biology. Given its close relationship to human, NHP podocytes may be more physiologically similar to its human equivalent.

Taken together, we have identified miR-27b as a key regulator of podocyte biology. MiR-27b regulates both podocyte survival and cytoskeleton changes upon injury, probably through targeting adora2b, a novel direct target of miR-27b. Our reports suggest that miR-27b/Adora2b axis play important roles in podocyte injury and could be potential therapeutic targets for podocyte protection in human renal diseases.

## Materials and methods

### Reagents

Puromycin aminonucleoside (Sigma, Cat#P7130); Phalloidin-iFluor^TM^ 594 Conjugate (AAT Bioquest, Cat# 23122); Paraformaldehyde (Sigma, Cat#158127–100 G); Collagen I (Thermofisher, Cat#A1048301);

### Glomeruli isolation and cell culture

Glomeruli from Sprague Dawley rats and Rhesus monkey were isolated using the sieving method. Briefly, kidney medulla was removed and only cortex was collected, which was further minced into ~1 mm pieces before collagenase digestion for 30 min at 37 °C. Digested tissues were passed through a series of sieves to collect glomeruli. Purified glomeruli were resuspended in podocyte culture medium and transferred to collagen coated flasks. Podocytes that grew out of the glomeruli were cultured in RPMI1640 medium (Gibco, Cat#C11875500BT) with 10% FBS and antibiotics. Culture medium was refreshed every other day. Primary podocytes were cultured for no more than 2–3 passages (within 2 weeks). For puromycin aminonucleoside (PAN) exposure assays 10 µg/ml PAN was added to the podocyte culture media 3 ~ 5 days before being collected for mRNA qRT-PCR analysis.

### miRNA TaqMan array analysis

miRNA expression profile analysis was done by using TaqMan® Array Human/Rodents MicroRNA A + B Cards Set v3.0 (Thermofisher) and following the manufacturer’s instructions. Total RNAs were extracted from glomeruli, tubule, and medulla tissues from mice, rats and non-human primates using trizol method and 1 µg total RNAs were used for RT reaction before running on TaqMan array.

### miRNA/siRNA transfection

NHP podocytes were transfected with miRNA/siRNAs using Lipofectamine RNAiMax (Invitrogen, Cat#13778030) following manufacturer’s suggestions. In brief, 50 nM miRNA/siRNA was transfected to NHP podocytes and total RNAs were isolated 2 days post transfection for downstream evaluations. miRNA mimics, inhibitors and siAdora2b were purchased from Dharmacon and RIBOBIO.

### Construction of miR sponge vectors

The sponge sequences were synthesized at Qinglan Biotech. The sequence is as follows: 5′-GCAGAACUUCGGACUGUGAAccggGCAGAACUUCGGACUGUGAAccggGCAGAACUUCGGACUGUGAAccggGCAGAACUUCGGACUGUGAAccggGCAGAACUUCGGACUGUGAAccggGCAGAACUUCGGACUGUGAA-3′.

We cloned oligonucleotides for microRNA binding sites with 4-nt spacers for bulged sites into pCMV-MCS-EF1-copGFP vector (System Biosciences, Cat# LV100A-1) digested with Sal I.

### Western blotting

Total protein lysate from NHP podocytes was prepared using RIPA buffer (PIERCE, Cat# 89900). Precast 4–12% gels were used for SDS-PAGE (Invitrogen, Cat# NP0322PK2) and transferred to cellulose membranes using Semi-dry system (BioRad, Cat# 170–3940). Membranes were blocked with 5% milk in TBST for at least 1 h at room temperature or overnight at 4 °C. Primary antibody was incubated for 1 h followed by dye-labeled secondary antibody for 45 min at room temperature. Membranes were then washed three times with TBST before imaging analysis using LI-COR system. Primary antibodies included anti-Adora2b (NHP) (LifeSpan Bioscience, Cat#LS-A680), anti-Adora2b (Rat) (Millipore, Cat#AB1589P), anti-Tubulin (Thermal Fisher, Cat#MA1-19401), anti-Gapdh(Santa Cruz, Cat#SC-25778) anti-Nephrin (Abcam, Cat#ab85379), anti-Synaptopodin (Abcam, Cat#101883), anti-WT-1 (Epitomics, Cat#AC-0115), anti-cleaved Caspase3 (Cell Signaling, Cat#9661S), anti-Caspase-3 (Cell Signaling, #9662), and anti-Actin (SCB, #SC-1616).

### Immunostaining

Cells were washed twice with PBS and fixed with 4% paraformaldehyde at room temperature for 20 min. Fixed cells were permeabilized with 0.1% Triton X-100 for 5 min. Cells were then blocked in 5% BSA in PBS containing 0.1% Triton X-100 for 1 h at room temperature. Primary antibody was diluted from 1:100 to 1:400 in 2.5% BSA PBS containing 0.1% Triton X-100, according to the manufacturer’s suggestion. Cells were stained with primary antibody for 1 h and then washed three times with PBS. Secondary antibody was diluted 1:400 and cells were stained for 45 min at room temperature. To stain the cytoskeleton, podocytes were incubated with Phalloidin-iFluor^TM^ 594 Conjugate (AAT Bioquest, Cat# 23122) for 60 min at room temperature.

### Dual luciferase assay

Adora2b 3′-UTR (Cyno Macaque) was cloned into pmiR-glo vector (Promega, Cat#E1330). A total of 20 ng vectors were transfected into 4000 293FT cells in 96-well plates together with miRNA mimics and inhibitors. Cells were collected 2 days after transfection and assayed by using Dual-Glo luciferase assay system (Promega, Cat# E2920). Assay procedure was the same as suggested by the manufacturer.

### Cell viability test

A total of 2000–4000 NHP podocytes were seeded in each well of 96-well plates. PAN treatment was usually started on the next day. Five days post PAN treatment, the medium was changed to 100 µl substrate mix (80 µl of culture medium + 20 µl substrate (Promega, Cat#G3582)). The cells were incubated with the substrates for 1 h at 37 °C and cell viability was measured by absorbance reading at 490 nm.

### Flow cytometry analysis

Trypsinized single cells were washed twice with cold PBS and resuspended in 1X Binding buffer from Apoptosis Detection Kit (BD Biosciences, Cat#559763). PE-Annexin V and 7-AAD were then added and incubated with the cells for 15 min at room temperature. Extra amount of binding buffer was then added to dilute the samples and FACS analysis was done within 1 h.

### In situ hybridization

FFPE kidney slides were incubated in 65 °C oven for 1 h the day before ISH and then stored in 4 °C for overnight. Slides were then deparaffinized with a series of xylene and ethanol washes and soaked in PBS in the end. Tissue sections were treated with 10 µg/ml Protease K for 20 min before washing with PBS and dehydrated with 70–99% ethanol washes. LNA-miR probes were added onto tissue sections and incubated for 1 h at 55 °C. The sections were further washed with several times with 5XSSC, 1XSSC, and 0.2XSSC before blocking with blocking buffer for 15 min at room temp. Anti-DIG antibody was then applied for 1 h at room temp and washed three times with PBST. Finally, the slides were exposed to AP substrates for 2 h at 30 °C before stopping the reaction and counter stain with Nuclear Fast Red. The slides were then dehydrated and mounted using Eukitt mounting medium.

### Quantitative PCR

A total of 500–1000 ng of total RNAs were used for mRNA qRT-PCR reactions. Total RNAs were reverse transcribed with cDNA RT kit (Life Technologies, Cat# 4368814). All the primers were listed in supplemental tables. SyBr Green mix (Bio-Rad, Cat# 172–5121) was used for most of qPCR assays except listed as taqman assays. Bio-Rad CFX 384 system was used for all the qPCR analysis. For miRNA qPCR, 500–1000 ng of total RNAs were used for miRNA RT reaction following manufacturer’s suggestions.
